# Guanidinylation of the cold shock protein YB‐1: Molecular basis, structural changes and Notch‐3 receptor binding

**DOI:** 10.1002/pro.70188

**Published:** 2025-06-25

**Authors:** Anna Leitz, Batuhan Kav, Xiyang Liu, Hebah Fatafta, Vera Jankowski, Bastian Aggeler, Yingying Gao, Ina Verena Martin, Kristian Vogt, Rafael Kramann, Tammo Ostendorf, Thomas Rauen, Birgit Strodel, Ute Raffetseder

**Affiliations:** ^1^ Department of Nephrology and Clinical Immunology RWTH Aachen University Aachen Germany; ^2^ Institute of Biological Information Processing: Structural Biochemistry Forschungszentrum Jülich Jülich Germany; ^3^ Institute for Molecular Cardiovascular Research (IMCAR) RWTH Aachen University Aachen Germany

**Keywords:** guanidinylation, Jagged‐1, Notch‐3, receptor‐ligand interaction, YB‐1, YB‐1‐2G

## Abstract

Posttranslational modifications of Y‐box binding protein (YB)‐1 are the prerequisite for its very different protein functions. Here, we investigate the underlying molecular mechanisms of YB‐1 guanidinylation and link increased serum urea levels as well as the activity of glycine amidinotransferase (GATM) with guanidinylation. Computer simulations show changes in stability and conformation of the YB‐1 protein induced by these modifications. In particular, the secondary structure of the doubly guanidinylated YB‐1 (YB‐1‐2G) shows a reduced tendency to form *β*‐sheets, and the modified cold shock domain is more exposed to the solvent. Protein–protein docking techniques in conjunction with molecular dynamics simulations confirm the binding between YB‐1 and its receptor Notch‐3 at EGF domains 17–24 but show no significant differences in the binding behavior of YB‐1 and YB‐1‐2G. This is confirmed in two different types of receptor‐ligand binding assays. In addition, we demonstrate for the first time a high‐affinity binding of YB‐1 to another ligand binding site on the Notch‐3 receptor, thereby achieving effective displacement of the canonical ligand Jagged. In conclusion, we identified molecular processes that lead to the guanidinylation of YB‐1 and revealed their effects on the structure and binding to receptor Notch‐3.

## 1 INTRODUCTION

The highly conserved cold shock protein YB‐1 takes on multiple functions inside and outside the cell, both in homeostasis and in the course of disease (Hermert et al., 2020; Lindquist & Mertens, 2018; Raffetseder et al., 2012; Rauen et al., 2016; Tacke et al., 2014; Wang et al., 2016; Wang et al., 2017; Wang et al., 2023). It is becoming increasingly clear that YB‐1 is involved in numerous DNA‐ and RNA‐mediated processes in cells, such as DNA replication, repair, and transcription as well as mRNA splicing, stability, and translation (Chen et al., 2000; Dutertre et al., 2010; En‐Nia et al., 2005; Raffetseder et al., 2003; Wang et al., 2016; Wang et al., 2023). In addition, YB‐1 was found to be actively secreted (Frye et al., 2009) and is part of the neutrophil extracellular trap (NET) formation process (Wang et al., 2023). Serum YB‐1 levels may be potentially utilized as a biomarker for inflammation and cancer (Breitkopf et al., 2020; Ferreira et al., 2017; Frye et al., 2009; Rauen et al., 2009).

Human YB‐1 is a 324 amino acid long protein consisting of three domains: the N‐terminal A/P domain (residues 1–50), which is rich in prolines and alanines; the evolutionarily conserved cold‐shock domain (CSD) (51–129), which consists of five *β*‐sheets packed antiparallel to a *β*‐barrel (a so‐called OB‐fold topology); and the C‐terminal domain (130–324), which is mostly disordered (Chen et al., 2019; Didier et al., 1988; Eliseeva et al., 2011; Heinemann & Roske, 2021; Kloks et al., 2002; Lyabin et al., 2014). Various functions of the protein are mediated by post‐translational modifications (Alidousty et al., 2014; Breitkopf et al., 2020; Evdokimova et al., 2006; Hanssen et al., 2011; Shah et al., 2021; Sutherland et al., 2005). In the sera of patients with systemic lupus erythematosus (SLE) as well as in a lupus‐prone mouse model (MRL.*lpr*), we were able to demonstrate yet unknown post‐translational guanidinylations at two closely located lysines (K53, K58) within the CSD of YB‐1 (YB‐1‐2G) (Breitkopf et al., 2020). Double guanidinylation of K53 and K58 has been identified in patients with lupus nephritis, suggesting an association between this YB‐1 modification and renal involvement in this disease (Breitkopf et al., 2020).

Biochemically, the guanidinylation of a lysine residue represents a conversion into a homoarginine group (hArg). The enzyme glycine amidinotransferase (GATM), also called L‐arginine: glycine amidinotransferase (AGAT), catalyzes the formation of hArg from L‐lysine (Lys) (Davids et al., 2012; Ryan et al., 1969), which is characterized by an extension of the side chain (mass increase by 42 Da) and an increased basicity of the group (pKa value for lysine: 10.8; for homoarginine: 12.3). However, it is not yet clear whether the modification of lysine to homoarginine within the YB‐1 protein is achieved by GATM.

We hypothesize that this structural change in the highly conserved cold shock domain of YB‐1 is related to changes in its biological functions. For YB‐1‐2G, for example, changes in relation to its stability, the subcellular/extracellular localization, and/or the binding properties to its receptor Notch‐3 (Rauen et al., 2009) are conceivable. As previously shown, secreted, non‐guanidinylated YB‐1 has a pro‐inflammatory role (Frye et al., 2009; Hanssen et al., 2013). Our previous data indicate that the guanidinylated form of YB‐1 has anti‐inflammatory effects in T lymphocytes from lupus patients (Breitkopf et al., 2020). While extracellular, non‐guanidinylated YB‐1 represses the transcription of anti‐inflammatory interleukin (IL)‐10, YB‐1‐2G leads to a significant induction of IL‐10 mRNA expression, together with simultaneous down‐regulation of the expression of the membrane‐bound ligand Jagged‐1 in immune cells (Breitkopf et al., 2020). The molecular mechanism underlying the different behavior of the two YB‐1 forms remains to be elucidated.

Within the extracellular part of Notch‐3, several ligand‐binding regions have been described, including the classical ligand‐binding domain at the epidermal growth factor (EGF) repeats 10 and 11 (Joutel et al., 2004; Peters et al., 2004). In addition to this, the EGF domains 7–10, known to bind canonical ligands such as Jagged (Joutel et al., 2004; Peters et al., 2004), and 13–33, especially 20–23, for the non‐canonical ligand YB‐1 have been identified (Gera & Dighe, 2018; Rauen et al., 2009).

In this study, we employ a combination of *in‐silico*, in vitro, and cell‐based strategies to investigate the molecular aspects of YB‐1 guanidinylation and its effects on structure, receptor binding, stability, and cellular localization of the protein.

## 2 RESULTS

### 2.1 Overexpression of GATM in tubular cells leads to increased expression, secretion, and guanidinylation of YB‐1

**FIGURE 1:**
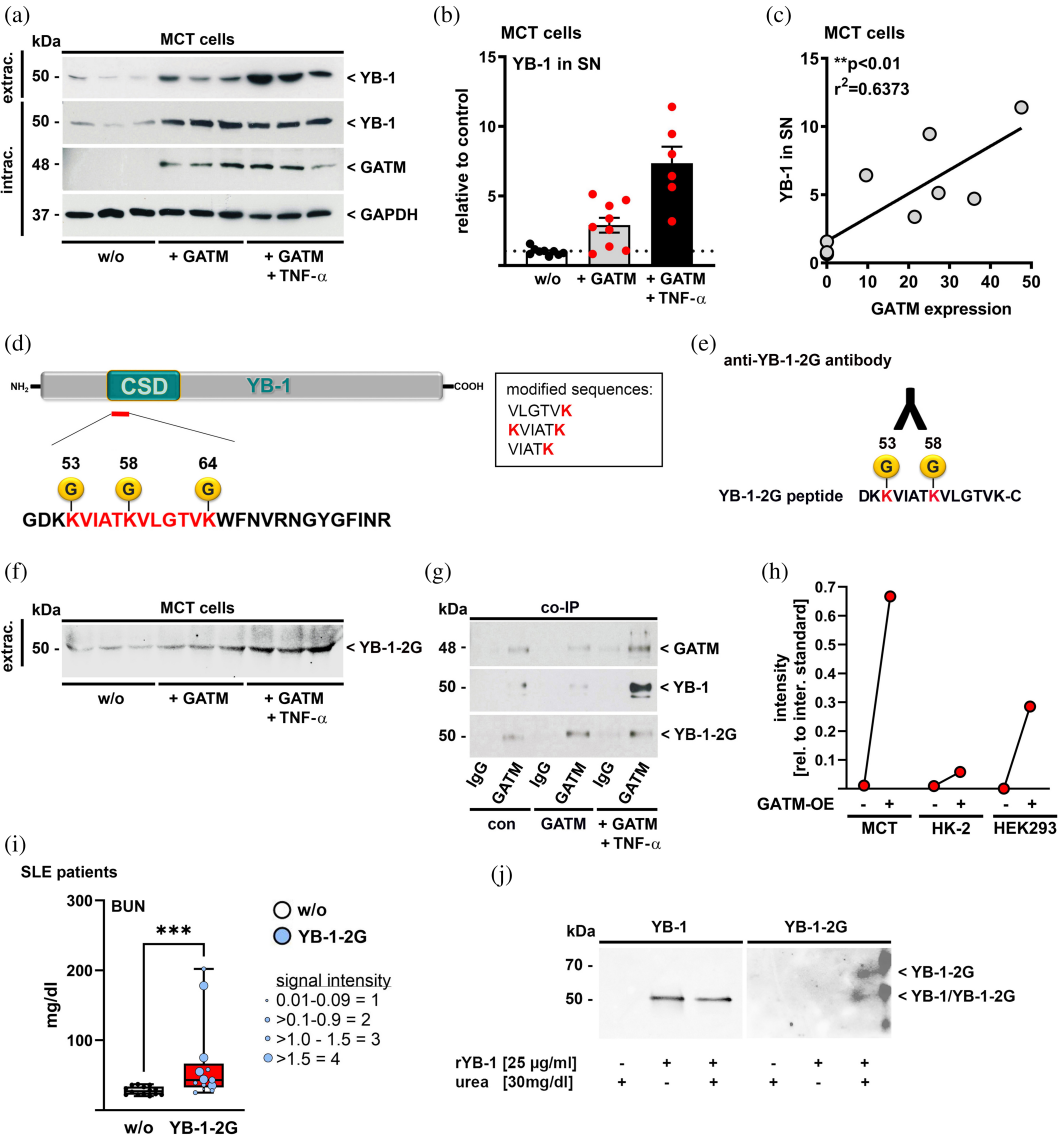
Underlying mechanisms of guanidinylation of YB‐1. (a) Immunoblots of MCT cell supernatants (extrac. YB‐1: Upper blot stripe) and cell lysates (intrac.: Stripe 2–3) for YB‐1, GATM and GAPDH (as loading control). The cells were previously transfected with GATM or control plasmid and additionally stimulated with TNF‐*α* (20 ng/mL, 2 h) or left untreated. Cells from each approach were individually transfected. (b) Quantification of band intensities of YB‐1 protein in supernatant (SN) relative to control (GAPDH). Data are presented as mean ± SEM. (c) Relation of GATM expression and YB‐1 in the SN based on band intensities of the immunoblot. (d) Sketch of YB‐1 and the sequence region (red) with three guanidinylated (G) lysines within the cold shock domain, found in the cell culture supernatants using mass spectrometry. The individual peptides and their modifications are listed in the box on the right. (e) A polyclonal peptide‐derived antibody against a dual guanidinylated YB‐1 was generated in rabbits. (f) Guanidinylated YB‐1 was detected in an immunoblot using SN of MCT cells. (g) Immunoblots of MCT cell lysate proteins immunoprecipitated by an anti‐GATM antibody developed with anti‐GATM, ‐YB‐1 and ‐YB‐1‐2G antibodies, respectively, show co‐immunoprecipitation of GATM with YB‐1 and YB‐1‐2G. (h) Guanidinylation mass‐signal intensity relative to the internal standard ^13^C‐ANG II after overexpression (OE) of GATM in different cell types. (i) The urea content in the serum of SLE patients (*n* = 28) with and without YB‐1‐2G (determined by mass spectrometry). The amount of YB‐1‐2G was estimated and is shown here: The size of the respective symbol represents the signal intensity relative to the internal standard ^13^C‐ANG II (1 = 0.01–0.09; 2 = 0.1–0.9; 3 = 1.0–1.5; 4 = >1.5). (j) In vitro guanidinylation of YB‐1 via urea. Detection of guanidinylated recombinant (r) YB‐1 after incubation with urea (30 mg/dL) by immunoblotting with anti‐YB‐1‐2G antibody. Total YB‐1 detected by anti‐YB‐1 C‐terminal antibody, served as comparison. CSD, cold shock domain; extrac., extracellular; G, guanidinylation; GAPDH, glycerinaldehyd‐3‐phosphat‐dehydrogenase; GATM, glycine amidinotransferase; intrac., intracellular; IP, immunoprecipitation; HK, human kidney, HEK, human embryonic kidney; MCT, mouse cortical tubule; SLE, systemic lupus erythematosus; TNF, tumor necrosis factor; YB‐1‐2G, guanidinylated YB‐1 detected by the anti‐YB‐1‐2G antibody.

To investigate a potential relationship between YB‐1 and GATM (EC 2.1.4.1), we focused on tubular cells due to GATM's high expression in renal tubules (Forst et al., 2021; Reichold et al., 2018). Cell culture experiments showed increased intracellular YB‐1 expression and secretion after GATM overexpression and even more in the combination of GATM overexpression plus tumor necrosis factor (TNF)‐*α* stimulation (Figure 1a–c). After GATM overexpression and TNF‐*α* stimulation, cell culture medium was analyzed for YB‐1‐2G by mass spectrometry. Three guanidinylated lysines were identified within the highly conserved CSD (Figure 1d) by characteristic mass fingerprint spectra of tryptic digested YB‐1 that were validated by MS/MS spectra. Especially, YB‐1 modification at lysines 53 and 58 (K53/K58) was found in sera from patients with lupus nephritis (Breitkopf et al., 2020). By using an antibody against a YB‐1‐2G peptide (Figure 1e, antibody specificity shown in Suppl. Figure 1A/B) carrying these two modifications, we were able to identify YB‐1‐2G in the supernatant of MCT cells after overexpression of GATM plus stimulation with TNF‐*α* (Figure 1f), without any significant change in molecular weight compared to YB‐1. TNF‐*α* alone did not result in significant YB‐1 or YB‐1‐2G secretion (Suppl. Figure 1C).

The GATM results could be confirmed in another tubular cell line, namely HK‐2 cells (Suppl. Figure 1D–F), where GATM overexpression resulted in a decrease in intracellular YB‐1 (Suppl Figure 1D), accompanied by a corresponding increase in extracellular YB‐1 (Suppl Figure 1E) and YB‐1‐2G (Suppl Figure 1F). A direct interaction between YB‐1 and GATM was detected by co‐immunoprecipitation using an anti‐GATM antibody and a nonspecific IgG as a control, respectively. Endogenous and overexpressed GATM was immunoprecipitated by the antibody (Figure 1g, upper stripe). Total (Figure 1g, middle stripe) and guanidinylated YB‐1 (bottom stripe) was co‐immunoprecipitated together with GATM, demonstrating a direct interaction of the two proteins. In line with this, the guanidinylation mass‐signal intensity of YB‐1 increased in three different renal epithelial cell lines (epithelial and embryonic) upon overexpression of GATM (Figure 1h).

### 2.2 SLE patients with YB‐1‐2G in serum have significantly higher urea concentrations, and urea can cause guanidinylation of YB‐1 in vitro

An alternative way to guanidinylate proteins is a biochemical process in the presence of urea (Schunk et al., 2021). A comparison of urea concentration with the presence of YB‐1‐2G in serum of 28 SLE patients showed that patients who had YB‐1‐2G in their serum had significantly higher blood urea nitrogen (BUN) levels (Figure 1i), with a direct correlation between the intensity of the guanidinylation signal and urea concentration (Suppl. Figure 1G). Incubation of recombinant (r) YB‐1 with urea resulted in increased guanidinylation of the protein (Suppl. Figure 1H–J), with even low urea concentrations (30 mg/dL) being sufficient (Figure 1j). In the negative control without urea, no guanidinylation of YB‐1 was detected (Figure 1j).

In summary, guanidinylation of YB‐1 can be achieved enzymatically by GATM or chemically by urea. In tubular cells, GATM binds and modifies YB‐1, which is subsequently found outside the cell.

### 2.3 Protein–protein docking of YB‐1 and YB‐1‐2G to EGF domains 17–24 within Notch‐3

**FIGURE 2:**
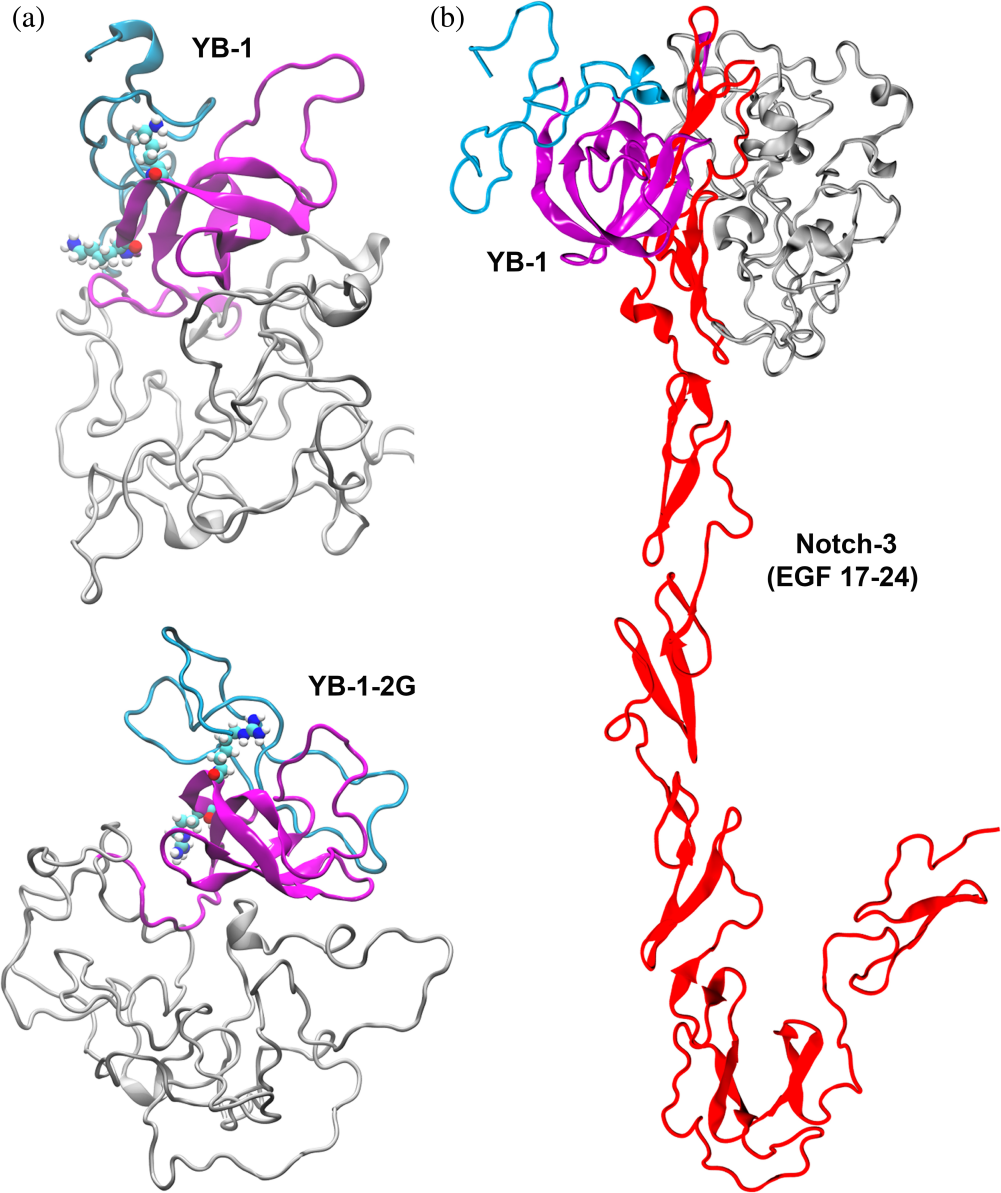
Protein–protein docking of YB‐1/YB‐1‐2G to EGF 17–24. (a) The most likely structures of YB‐1 (top) and YB‐1‐2G (bottom) as obtained from MD simulations applied to five different protein models obtained from the Robetta server are shown. Other likely YB‐1(−2G) structures are provided in Suppl. Figure 2. The proteins are shown as cartoon with the CSD colored magenta, the N‐terminal A/P domain cyan, and the C‐terminal domain in gray. The side chains at positions 53 and 58 are shown with atomic resolution (C atoms in cyan, N in blue, O in red, H in white). (b) The most likely YB‐1/EGF 17–24 complex structure obtained from docking followed by MD simulations. Both proteins are shown in cartoon with the same coloring for YB‐1 as used in (a) and the EGF repeats colored red.

We next used structural modeling to investigate the effects of guanidinylation on the interactions between YB‐1 and EGF repeats 17–24 of Notch‐3, a previously described YB‐1 binding site within the receptor (Rauen et al., 2009). To obtain possible binding poses, we used a consensus approach by employing three different protein–protein docking servers, FRODOCK (Ramírez‐Aportela et al., 2016), ZDOCK (Pierce et al., 2014) and HDOCK (Yan et al., 2020). The structure of EGF 17–24 subjected to docking was taken from the structure of the entire Notch‐3 receptor available in the AlphaFold database (Varadi et al., 2024) (entry number Q9UM47), while for YB‐1 and YB‐1‐2G, we used the five most probable structures identified by molecular dynamics (MD) simulations (discussed in detail below, Figure 2a and Suppl. Figure 2A–E). Analysis of the 150 candidate structures for the YB‐1(−2G)/EGF 17–24 complex revealed that YB‐1(−2G) binds to EGF 17–24 with similar probability via the ordered CSD or the disordered C‐terminal domain (Suppl. Table S1). For further refinement of the docking poses by MD simulations, we selected 5 complex structures for YB‐1 and five for YB‐1‐2G (applying the selection criteria provided in “Materials and Methods”) and simulated each of them six times: 3 × 50 ns with the YB‐1 variant used in docking and 3 × 50 ns, where we had transformed YB‐1 into YB‐1‐2G in the YB‐1 complexes and YB‐1‐2G into YB‐1 in the YB‐1‐2G complexes. The latter was performed to assess whether the stability of the complexes was affected by the modifications, which was found not to be the case. However, under all conditions, significant conformational flexibility was observed for EGF 17–24, leading to deviations from the initial extended domain arrangement (visible in the representative complex structures shown in Suppl. Figure 3A), which is consistent with other simulation and nuclear magnetic resonance studies for EGF (Kaplan et al., 2016; Martin‐Fernandez et al., 2019). In one of the complexes examined here, EGF 17–24 remained in its linear rearrangement, which is shown in Figure 2b.

**FIGURE 3:**
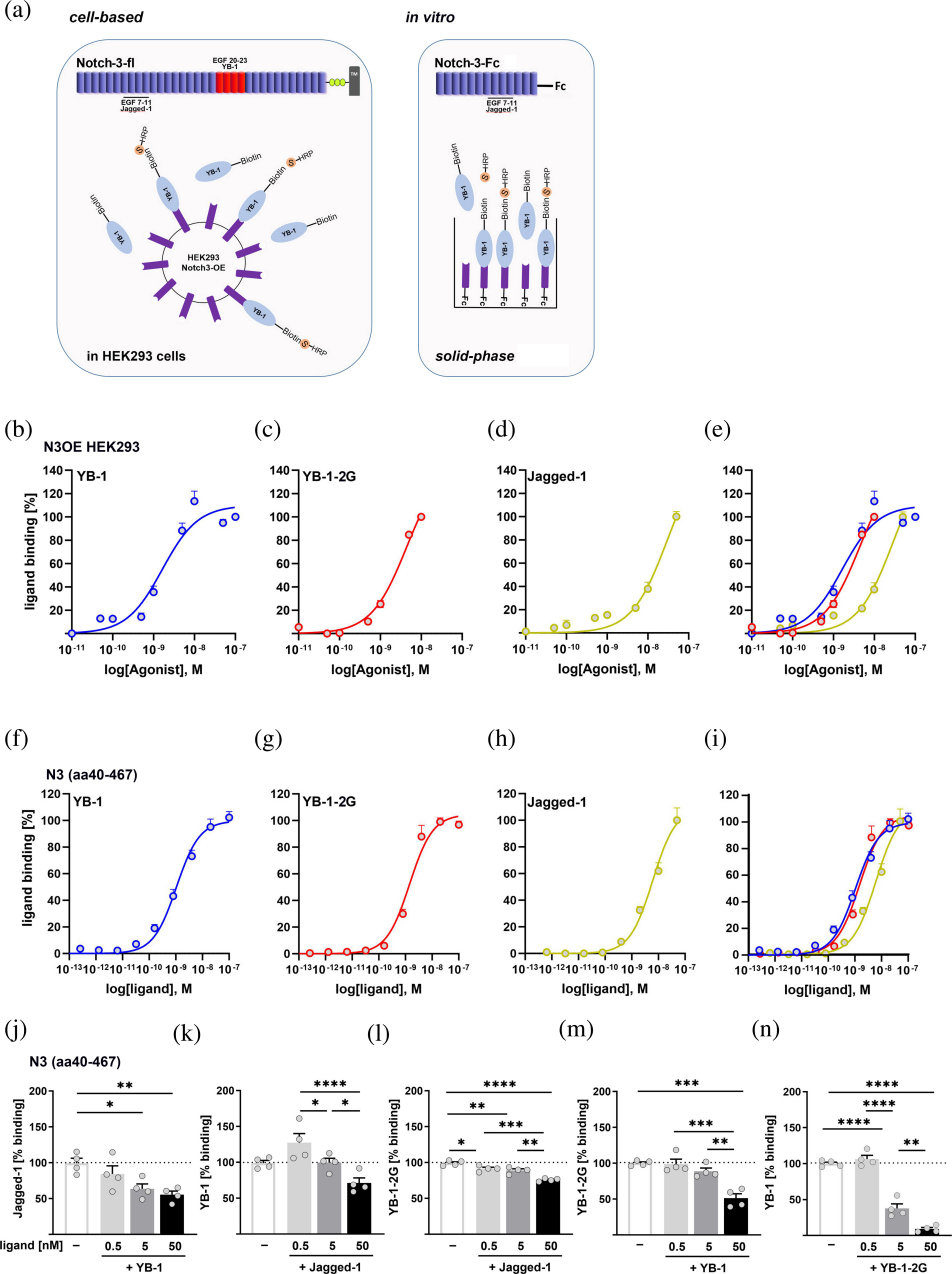
Binding and competition for binding of YB‐1, YB‐1‐2G, and Jagged‐1 to the receptor Notch‐3. (a) Illustration of the ligand binding assays in HEK293 cells after overexpression (OE) of Notch‐3 (left) and in the solid‐phase binding assay (right) to analyze the concentration‐dependent binding of biotinylated ligands by streptavidin (S)‐horseradish peroxidase (HRP). The receptor constructs with the included ligand binding domains for Jagged‐1 (EGF7‐11) (Joutel et al., 2004; Lin et al., 2010; Peters et al., 2004) and YB‐1 (EGF20‐23) (Rauen et al. 2009, Gera & Dighe 2018) are shown above, respectively. (b)–(e) Overexpression of full‐length Notch‐3 receptor in HEK293 cells enables high‐affinity binding of YB‐1 (b), YB‐1‐2G (c) and Jagged‐1 (d). Overlay of the curves (e). (f)–(i) Solid‐phase binding assay to analyze the concentration‐dependent binding of YB‐1 (f), YB‐1‐2G (g), and Jagged‐1 (h) to the part of the extracellular domain of the Notch‐3 receptor (Ala40–Glu467) that includes the EGF domains responsible for Jagged‐1 binding (EGF 7–11). (i) Overlay of curves. (j)–(n). Competition analyzes for binding to the Notch‐3 receptor for YB‐1 versus Jagged‐1 (j), Jagged‐1 versus YB‐1 (k), Jagged‐1 versus YB‐1‐2G (l), YB‐1 versus YB‐1‐2G (m) and YB‐1‐2G versus YB‐1 (n). The percentage of maximum binding at the highest measured ligand concentration is shown; the non‐specific binding of the respective ligand is subtracted. Data are presented as mean ± SEM. EGF, epidermal growth factor; N3OE, Notch‐3 overexpression; aa, amino acid.

To monitor the stability, the number of contacts between YB‐1 (or YB‐1‐2G) and EGF 17–24 was calculated (Suppl. Figure 3B/C). We further quantified the change in the total number of contacts during the simulations relative to the number of contacts in the corresponding docking structure. The time‐ and MD run‐averaged changes are provided in Suppl. Table S2. No significant decrease in the number of contacts was observed for any of the complexes, indicating that the complexes are stable on the simulated time scale. We further found that switching from YB‐1 to YB‐1‐2G or from YB‐1‐2G to YB‐1 did not affect the stability of the complexes. The maximum loss in contacts was less than 2% for all but three cases, while the loss was below ~9% for the other three complexes. Additionally, an increase of ~33% and 24% in contacts was observed for complex 3 of YB‐1 and after mutating it to YB‐1‐2G/EGF 17–24, respectively. This suggests that this complex was not optimally predicted by docking. The finding that the complexes are stable did not change after extending the simulations for one complex per system to 100 ns.

### 2.4 YB‐1 and YB‐1‐2G bind to Notch‐3 with equal affinity and compete with the canonical ligand Jagged‐1

To compare the binding behavior of YB‐1 and its guanidinylated form in cells, we analyzed concentration‐dependent binding in HEK293 cells after overexpression of the full‐length receptor Notch‐3 (N3OE), which contains the described binding site of YB‐1 (Gera & Dighe, 2018; Rauen et al., 2009) (Figure 3a, left panel). YB‐1 and its guanidinylated form bound in the same concentration range (Figure 3b, c, e), but with higher affinity than the canonical ligand Jagged‐1 (Figure 3d/e).

To investigate whether YB‐1 or YB‐1‐2G can also bind to the well‐characterized Jagged‐1 binding site (Joutel et al., 2004; Lin et al., 2010), we used a truncated Notch‐3 receptor construct (aa40‐467). This construct includes EGF domains 10 and 11, which are essential for the interaction with canonical ligands (Joutel et al., 2004; Peters et al., 2004), but lacks EGF domains 20–23 (Figure 3a, right panel) that have previously been identified for YB‐1 (Gera & Dighe, 2018; Rauen et al., 2009).

A concentration‐dependent, high‐affinity binding was demonstrated for both YB‐1 and for the in vitro guanidinylated form. Binding behavior of the unmodified (Figure 3f) and the guanidinylated (Figure 3g) form of YB‐1 was indistinguishable, and both tended to have a slightly higher affinity than the canonical ligand Jagged‐1 (Figure 3h, i).

We next asked whether there is direct competition between YB‐1 and Jagged‐1 for binding to receptor Notch‐3. The Jagged‐1 binding could be displaced by YB‐1 in a concentration‐dependent manner (Figure 3j, at highest concentration to 56% ± 5.0% of binding) and vice versa, the binding of YB‐1 (Figure 3k) as well as YB‐1‐2G (Figure 3l) could be displaced by Jagged‐1, although not quite as efficiently (72.4% ± 7.1% and 76.68% ± 1.0%, respectively). However, YB‐1 was able to efficiently displace YB‐1‐2G from Notch‐3 (Figure 3m; 51.3% ± 6.2%) and vice versa (Figure 3n; 9.1% ± 4.5%).

Taken together, YB‐1 and YB‐1‐2G bind to Notch‐3 with comparable affinity and compete with the canonical ligand Jagged‐1 for its binding.

### 2.5 Guanidinylation leads to a change in the solvent‐accessible surface of YB‐1‐2G

**FIGURE 4:**
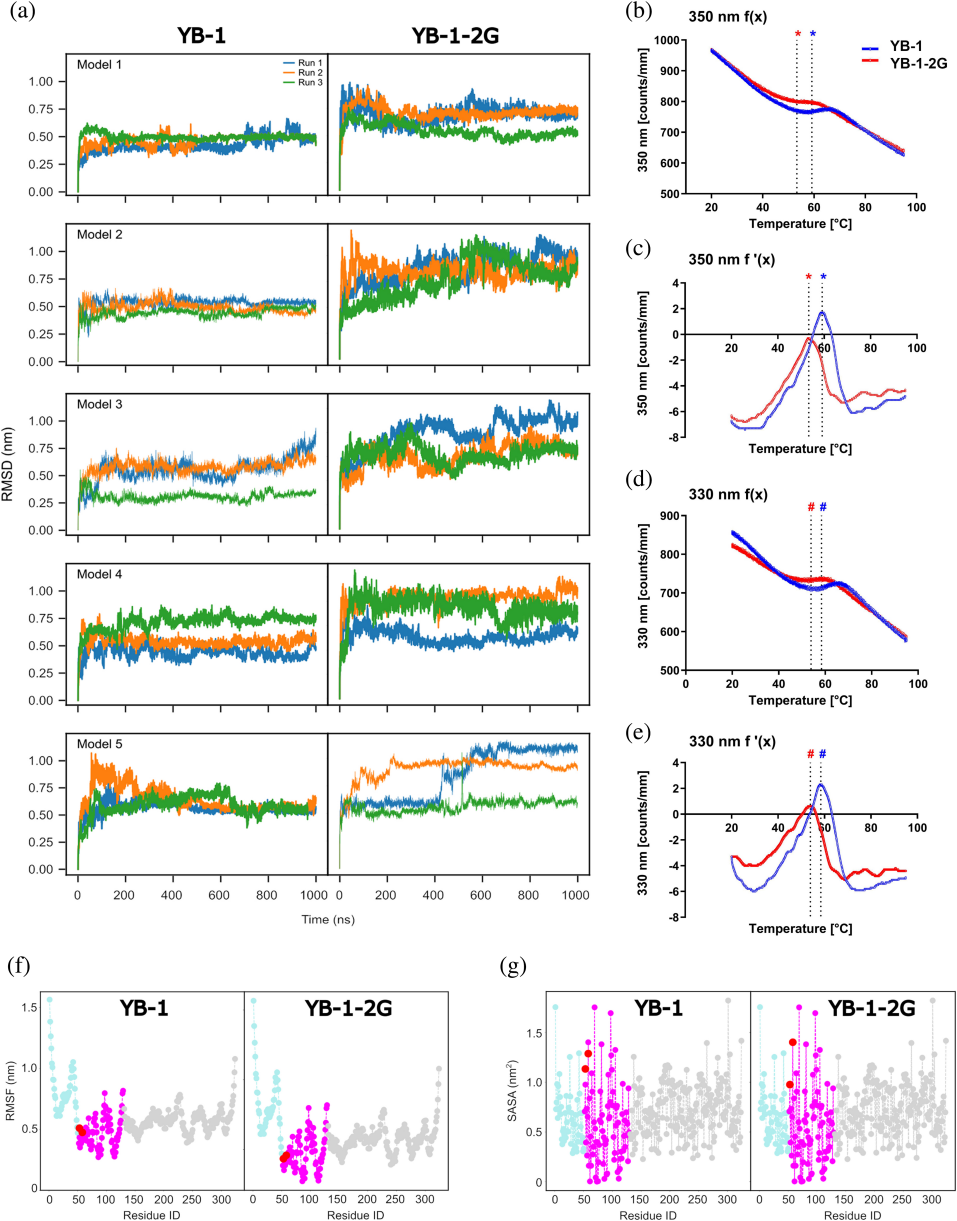
MD simulations and NanoDSF measurements of YB‐1 and YB‐1‐2G. (a) The RMSD during the 3 × 1 μs MD simulations performed for the five YB‐1 (left) and YB‐1‐2G models (right) determined by Robetta. (b)–(e) NanoDSF measurement to assess stability of YB‐1 and YB‐1‐2G. (b) The resulting curve (*f*(*x*)) was obtained by plotting the counts/mm at a wavelength of 350 nm, at which tyrosine absorbs, against the temperature. The inflection points indicate the melting temperatures of YB‐1 (blue asterisk; 59.16°C) and YB‐1‐2G (red asterisk; 53.35°C). (c) The first derivative (*f*′(*x*)) of the curve obtained at 350 nm. The maximum values indicate the melting temperatures of YB‐1 and YB‐1‐2G. (d) Curve (*f*(*x*)) obtained when plotting the counts/mm at a wavelength of 330 nm, at which tryptophan absorbs against the temperature. The inflection points indicate the melting temperatures of YB‐1 (blue hash tag; 58.4°C) and YB‐1‐2G (red hash tag; 53.9°C). (e) The first derivative (*f*′(*x*)) of the curve obtained at 330 nm. The maxima correspond to the melting temperatures of YB‐1 and YB‐1‐2G. (f) The model‐ and time‐averaged RMSF per residue of YB‐1 and YB‐1‐2G (cyan: A/P domain, magenta: CSD, gray: C‐terminal domain, red dots: Positions 53 and 58). (g) The model‐ and time‐averaged SASA per residue of YB‐1 and YB‐1‐2G, with the same coloring as in (f).

To detect possible structural differences between YB‐1 and YB‐1‐2G, we performed simulations. We started by generating five structural models for YB‐1 using Robetta, which produced a structurally conserved CSD structure, while the A/P‐ and C‐terminal domains differ in their internal structure and orientation relative to the CSD. The YB‐1‐2G structures were generated from the YB‐1 models by introducing the guanidinylated lysines at positions 53 and 58. The structural dynamics of YB‐1 and YB‐1‐2G were assessed by MD simulations. The most frequently sampled conformations indicated notable structural fluctuations (Suppl. Figure 2A–E). To quantify these fluctuations, we calculated the root mean square deviation (RMSD) as a function of simulation time (Figure 4a), which shows on the one hand that all 30 MD simulations converged within the simulated time, but on the other hand that the final deviations are considerable, with values between 0.5 and 0.8 nm. Moreover, it seems that guanidinylation leads to somewhat larger fluctuations, indicating reduced protein stability for YB‐1‐2G compared to YB‐1.

To further assess the protein stability, nano differential scanning fluorometry (DSF) measurements were conducted showing higher stability for YB‐1 than for YB‐1‐2G. This is evidenced by the higher melting temperatures observed for YB‐1 at both 350 nm (Figure 4b/c) and 330 nm (Figure 4d/e). The melting temperatures for YB‐1 were recorded at 58.16°C (350 nm) and 58.4°C (330 nm), while the corresponding values for YB‐1‐2G were 53.35°C (350 nm) and 53.9°C (330 nm). This indicates that YB‐1‐2G is more susceptible to unfolding and therefore less stable.

To understand the origin of these deviations, we determined the root mean square fluctuations (RMSF) along the protein backbone (Figure 4f), which identified the CSD domain as the rigid part of the protein for both YB‐1 and YB‐1‐2G, while the A/P‐ and C‐terminal domains are more flexible and disordered without reaching a consensus structure, as shown by the simulated structures (Suppl. Figure 2A–E). No notable RMSF differences are observed between YB‐1 and YB‐1‐2G. We further analyzed the CSD domain by checking the transformation of the secondary structure during the MD simulations (Suppl. Figure 2A–E). This analysis demonstrated that for all models of the two YB‐1 variants, the *β*‐barrel topology is stable, but guanidinylation at sites 53 and 58, which are part of a *β*‐strand in YB‐1, leads to destabilization of the affected *β*‐sheet region, which consists of the region at the beginning of the CSD domain and the region spanned by residues 105–155. In addition, guanidinylation leads to a change in the solvent‐accessible surface of YB‐1‐2G, resulting in greater exposure of the CSD domain to solvent, particularly in the case of modified K58, which may make the protein more susceptible to interaction with other molecules (Figure 4g).

## 3 DISCUSSION

To date, there is only a little data available on the post‐translational mechanism of protein guanidinylation. Strikingly, guanidinylation of proteins occurs particularly in the context of kidney damage. For example, albumin guanidinylation was detected in patients with chronic renal failure, leading to a conformational change and altered transport properties of albumin (Rueth et al., 2015). Guanidinylated apolipoprotein C‐3 (ApoC3) increases the release of inflammatory cytokines and is associated with chronic kidney disease (CKD) and CKD‐associated vascular injury (Schunk et al., 2021). Post‐translational guanidinylation of YB‐1 is present in both lupus mouse models and in SLE patients (Breitkopf et al., 2020). In SLE patients, the double guanidinylation (YB‐1‐2G) has been found particularly in sera from patients with renal impairment, namely lupus nephritis (Breitkopf et al., 2020).

However, the metabolic pathways responsible for the conversion of lysine to homoarginine are not yet fully understood. An enzymatic mediation by GATM, in which a guanidino group is transferred from arginine to lysine, has been described (Davids et al., 2012). We were able to show that a significantly higher intensity of the YB‐1‐2G mass signal is associated with the presence of GATM and that there is a direct interaction between the two proteins, which led to the subsequent secretion of YB‐1 from tubular cells. Strikingly, GATM is strongly expressed in renal tubule cells of the kidney and in polarized M2 macrophages (Forst et al., 2021; Wyss & Kaddurah‐Daouk, 2000; Yu et al., 2022). Macrophages are the dominant immune cell type, with CD163^+^ M2c‐like macrophages predominating in renal biopsies from patients with lupus nephritis (Olmes et al., 2016). Thus, this enzymatic mechanism of action seems very likely for the high YB‐1‐2G concentrations in lupus nephritis. However, so far only the enzymatic conversion of isolated glycines/lysines to homoarginine has been described, and future studies must clarify to what extent a lysine located within a protein can also be modified by the GATM enzyme and how exactly the secretion of the modified protein occurs.

Nevertheless, we and others (Schunk et al., 2021) have shown that urea can also cause guanidinylation of proteins in vitro (Figure 1j), which is particularly interesting because elevated BUN levels are common in CKD (Vanholder et al., 2018). Although only a few SLE patients had urea concentrations that were above the pathophysiologically relevant value >100 mg/dL (Figure 1i), the increased level of urea compared to the control subjects seems to be sufficient for the biochemical process of guanidinylation. Thus, we were able to demonstrate that the incubation of YB‐1 with a urea concentration already of 30 mg/dL leads to an increase in the guanidinylation of YB‐1.

The discovery of guanidinylation of YB‐1 raised the question of whether this modification alters the binding behavior to the receptor Notch‐3. Docking results clarified that the change from YB‐1 to YB‐1‐2G or from YB‐1‐2G to YB‐1 did not affect the stability of the complexes between YB‐1 and Notch‐3. This result can be explained by the observation that guanidinylation sites 53 and 58 are not in direct contact with EGF 17–24. The docking results were confirmed by two different types of binding studies, in cells and in a solid phase assay, in which the binding of the guanidinylated YB‐1 was indistinguishable from the unmodified form, but unexpectedly the binding had a higher affinity than that of the canonical ligand Jagged‐1.

In addition to the previously described YB‐1 binding site (EGF13‐33; aa517‐1367) within the Notch‐3 receptor (Rauen et al., 2009), we have, for the first time, demonstrated YB‐1 binding to upstream Notch‐3 domains (aa40‐467). This region includes the well‐described binding site for canonical ligands (EGF10/11) such as Jagged‐1 (Joutel et al., 2004; Peters et al., 2004), and both YB‐1 and YB‐1‐2G turned out to be potent competitors for Jagged‐1 binding at the receptor Notch‐3.

However, if binding to the receptor Notch‐3 is unchanged, the question remains as to what is responsible for the different functions of YB‐1 and YB‐1‐2G (Breitkopf et al., 2020). Several post‐translational modifications have been reported for YB‐1, with phosphorylation at Ser102 being the most prominent. This modification is associated with various functions, most of which are related to its intracellular localization (Alidousty et al., 2014; Sutherland et al., 2005). It has been shown that YB‐1 can be acetylated (Wu et al., 2015) and ubiquitinylated (Boeing et al., 2016) at lysine 64. We can only speculate that guanidinylation of YB‐1 at positions K53/58/64 prevents other post‐translational modifications, thereby altering the stability and functions of the protein as compared to the non‐guanidinylated but otherwise modified form. Other explanations are also provided by the results of the computer‐based structural analyzes of the proteins. The guanidinylation sites are on the *β*‐strand/bridge. While this modification does not alter the overall structure of the CSD, it does weaken the *β*‐sheet formed between this region and residues 105–115, reducing the overall protein stability.

In summary, despite their different functions, YB‐1 and YB‐1‐2G do not display altered binding capabilities to the Notch‐3 receptor. However, the lower stability and more exposed CSD of YB‐1‐2G may explain why it displays different molecular capabilities compared to unmodified YB‐1. The newly discovered binding site of YB‐1 at the Notch‐3 receptor, which functions competitively with the ligand Jagged‐1, has the potential to develop interesting therapeutic applications in the future.

## 4 MATERIALS AND METHODS

### 4.1 Cell culture

Human kidney (HK)‐2 and murine cortical tubular (MCT) renal epithelial cells were cultured as described (Hermert et al., 2020) and transfected with Lipofectamine™ (Thermo Fisher Scientific) according to the manufacturer's instructions. Human embryonic kidney cells (HEK293) were cultured and transfected as described before (Rauen et al., 2016). GATM‐transfected cells were stimulated for 2 h with 20 ng/mL murine or human recombinant TNF‐*α* (ImmunoTools).

Cells were transiently transfected with full‐length murine pcDNA‐Notch‐3 receptor (Karlstrom et al., 2002), human CD8‐GATM (Reichold et al., 2018), or corresponding empty vector. Supplements and media were purchased from Life Technologies. MCT and HEK293 cells were cultured in DMEM GlutaMAX™ supplemented with 10% FCS, 100 U/mL penicillin and streptomycin, unless otherwise stated. HK‐2 cells were cultured in DMEM supplemented with 20% FCS + 100 U/mL penicillin and streptomycin + 1% sodium pyruvate + 1% glutamine + 1% non‐essential amino acids (NEAA) + 10 μg/mL insulin. Cells were maintained at 37°C in humidified air with 5% CO_2_.

### 4.2 Crosslinking of antibodies to protein‐A/Sepharose beads for co‐immunoprecipitation

20 μg of antibodies were added to 300 μL Protein A‐Sepharose beads suspension (Life Technologies, Darmstadt) and incubated for 1 h at RT. The antibody‐bead suspension was centrifuged at 2500 rpm for 5 min, the supernatant was discarded, and the pellet was suspended in 1 mL of 0.2 M sodium borate solution (SBS, pH 9.0). The suspension was centrifuged at 2500 rpm for 5 min. The pellet was collected, washed with 1 mL of SBS, and 100 μL was retained as a pre‐coupling sample for later checking of coupling efficiency. Dimethyl pimelimidate was added as a solid (final concentration 20 mM) and the mixture was incubated at RT for 30 min. Another 100 μL was taken as a post‐coupling sample. The beads were collected by centrifugation and suspended in 1 mL of 0.2 M ethanolamine solution (pH 8.0), followed by centrifugation at 2500 rpm for 5 min. The pellet was collected and resuspended in 1 mL of 0.2 M ethanolamine solution and incubated at RT for 2 h. The beads were collected again by centrifugation and resuspended in 150 μL of 0.03% sodium azide/PBS and stored at 4°C. To check the coupling efficiency, the pre‐ and post‐coupling samples were taken up in 20 μL SDS sample buffer after centrifugation, separated in a 10% SDS gel, and then stained with Coomassie staining solution.

### 4.3 Co‐immunoprecipitation

Cell lysates (200 μg protein) and 40 μL Protein A‐Sepharose antibody suspension were combined with 500 μL immunoprecipitation (IPP) buffer (20 mM HEPES pH 7.4, 100 mM KCl, 5 mM magnesium acetate, 1 mM DTT, 0,025% Triton X‐100, proteinase inhibitor); the mix was incubated for 1.5 h at 4°C. The bead pellet was collected following 2500 rpm centrifugation for 5 min at 4°C, resuspended in IPP buffer, and washed eight times. The final wash was done in PBS buffer. After the washing steps, the Sepharose pellet was dissolved in 50 μL sample buffer for Western blot analysis.

### 4.4 Protein extraction and Western blot analysis

Cell lysis, sodium dodecyl sulfate (SDS) gel electrophoresis, and subsequent blotting were performed as previously described (Raffetseder et al., 2003). Anti‐GAPDH (NB300‐221, Novus Biologicals), anti‐GATM (12801‐AP, Proteintech), and anti‐human YB‐1 C‐terminal (Y0396, Sigma‐Aldrich) antibodies were used for the detection of specific proteins. A peptide‐derived affinity‐purified polyclonal anti‐YB‐1‐2G antibody was made by Davids Biotechnologie GmbH in rabbits based on the following peptide sequence GGDK(X)VIAT(X)VLGTVC (X = Homoarginine) (Peptide Specialties Laboratory GmbH). Ponceau S staining was performed to ensure uniform loading of extracellular proteins. To quantify band intensity, the image processing program ImageJ was used.

### 4.5 Mass spectrometry

Protein solutions, cell lysates, serum samples, and cell culture supernatants were prepared as described previously (Kork et al., 2018) and MALDI‐time of flight (TOF)/TOF MS (Ultraflex III; Bruker‐Daltonics, Bremen, Germany) MS was performed (Breitkopf et al., 2020), using ^13^C‐ANG II as the internal standard.

### 4.6 In vitro guanidinylation of YB‐1

For binding assays, 0.06 μg/μL recombinant YB‐1 (RPPB5170, Assay Genie) was incubated in 5 mM O‐methylisourea bisulfate solution (Sigma‐Aldrich) pH 11 for 6 h at 0°C. After that, the sample was rebuffered using Amicon™ Ultra‐2‐Centrifugal Filter Units (Sigma‐Aldrich) according to the manufacturer's instructions. The protein was reconstituted in PBS + 20% glycerol +0.1% BSA. Alternatively, 25 μg/mL recombinant YB‐1 (Assay Genie) was incubated in 30–200 mg/dL urea solution pH 11 for 6 h at 0°C. The success of the guanidinylation was tested via MALDI MS and Western blot (Suppl. Figure 1B, H–J). The protein concentration was determined with NanoOrange™ Protein Determination Kit (Thermo Fisher Scientific), according to the manufacturer instructions.

### 4.7 Ligand binding assays

Recombinant YB‐1 (rYB‐1, Assay Genie), rYB‐1‐2G, and rJagged‐1 (ab220536, Abcam) were biotinylated according to the manufacturer's instructions (Thermo Fisher Scientific). Excess biotin was removed using ZEBA™ Spin Desalting Columns (Thermo Fisher Scientific) and protein concentration was determined again.

For in vitro binding assays, 0.5 μg/mL Notch‐3‐Fc‐Chimera (aa40‐467, EGF 1–11; BioLegend) was diluted in carbonate buffer (15 mM Na_2_CO_3_, 35 mM NaHCO_3_, 0.2 g/L NaN_3_, pH 9.6) and coated on Nunc™ MaxiSorp™ microtiter plates (100 μL/well) at 4°C overnight. The plate was washed once with 300 μL binding buffer (50 mM Tris, 150 mM NaCl, 5 mM MnCl_2_, 0.1% BSA, pH 7.2) and blocked with 300 μL ROTI®Block (Carl Roth), diluted 1:10 in deionized water for 2 h at RT on an orbital shaker. In the next step, the plate was washed 2× with 300 μL binding buffer. This was followed by adding different concentrations of biotinylated rYB‐1 (Assay Genie), rYB‐1‐2G, and rJagged‐1 (Abcam) (10^−7^ – 10^−11^ mol/L, 100 μL/well) diluted in binding buffer and incubation at 4°C for 3 h. Subsequently, the plate was washed 3× with 300 μL ice cold binding buffer and incubated with 100 μL Pierce™ Streptavidin‐HRP high sensitivity (Thermo Fisher Scientific), diluted 1:40,000 in PBS + 0.05% TWEEN‐20 + 3% BSA for 1 h at RT on an orbital shaker. The plate was washed 5× with 300 μL PBS + 0.05% TWEEN‐20, followed by adding 100 μL TMB‐substrate (Becton Dickinson) to each well. As soon as the color reaction was sufficient, it was stopped using H_2_SO_4_ (30 μL/well) and the absorbance was measured at 450 nm in a microplate reader (SpectraMax® iD3, Molecular Devices).

For the cell‐based binding assays, 2 × 10^5^ full‐length murine pcDNA‐Notch‐3 receptor overexpressing HEK293T cells were seeded per well in 24‐well cell culture plates coated with 300 μL 0.1 mg/mL Poly‐D‐Lysin (Thermo Fisher Scientific) and allowed to attach overnight. Next, the cells were washed once with 1 mL binding buffer, and different concentrations of biotinylated rYB‐1 (Assay Genie), rYB‐1‐2G and rJagged‐1 (Abcam) (10^−7^ to 10^−11^ mol/L, 100 μL/well) diluted in binding buffer were added and incubated at 4°C for 3 h. The plate was washed 3× with 1 mL ice‐cold binding buffer and blocked with 1 mL SmartBlock™ (Candor) for 1 h at RT on an orbital shaker. Cells were subsequently washed two times with 1 mL PBS and incubated with 250 μL Pierce™ Streptavidin‐HRP high sensitivity (Thermo Fisher Scientific), diluted 1:10,000 in PBS + 3% BSA for 1 h at RT on an orbital shaker. The wells were washed 3× with 1 mL PBS and subsequently 250 μL TMB‐substrate were added to each well. As soon as the color reaction was sufficient, it was stopped using 2 N H_2_SO_4_ (125 μL/well) and the absorbance was measured at 450 nm.

### 4.8 Human sample collection

Biosampling was approved by the involved local ethic committee (EK 144/10) and in accordance with the principles of the Declaration of Helsinki. Written informed consent was obtained from all patients. The cohort studied included 28 SLE patients, from whom serum samples were analyzed for YB‐1‐2G using mass spectrometry.

### 4.9 Molecular dynamics simulations

MD simulations were performed for YB‐1 and YB‐1‐2G. Homology modeling using the Robetta web server (Baek et al., 2021) was used to obtain structural models as the starting point for the respective MD simulation, since only the CSD is available in structural databases. This yielded five structure models for YB‐1, which differ significantly in the disordered C‐terminal region, while the CSD structure is conserved among the models with a maximum RMSD of 0.4 nm between them. The alanine/proline (A/P) region varies from no well‐defined secondary structure (models 1, 2, and 5) to two *β*‐sheets (models 3 and 4). We used these five models as the starting structure for the MD simulations of both YB‐1 and YB‐1‐2G, where guanidinylation was achieved by mutating K53/K58 to hArg. Per starting structure and protein, we ran three independent 1000 ns MD simulations, adding up to 15 μs of simulation for YB‐1 and YB‐1‐2G. The MD simulations were performed with GROMACS 2021 (Abraham et al., 2016) in conjunction with the CHARMM36 force field (Huang & MacKerell Jr., 2013) or modeling the proteins and TIP3P as water model (Jorgensen et al., 1983). For modeling the hArg side chains, we generated the parameters by combining the existing CHARMM36 parameters for Lys and Arg side chains. The temperature was set to 25°C to mimic the experimental conditions using the Nosé–Hoover thermostat (Hoover, 1985; Nose, 1984) and the pressure to 1 bar using the Parrinello–Rahman barostat (Parrinello & Rahman, 1981). The MD simulations involved periodic boundary conditions, entailing the usage of the particle mesh Ewald method (Darden et al., 1993) to calculate the electrostatic interactions. The non‐bonded interactions computed in real space were considered up to a cutoff value of 1.2 nm. All lengths of bonds to hydrogen atoms were constrained using the LINCS algorithm (Hess et al., 1997), allowing for the use of a 2 fs time step in the MD simulations.

### 4.10 Protein–protein docking

Protein–protein docking was applied to model the binding of YB‐1 and YB‐1‐2G to the Notch‐3 receptor. To this end, the five most prominent structures of YB‐1 (YB‐1‐2G) resulting from the MD simulations were docked to the AlphaFold 2 (Jumper et al., 2021) structure of the EGF repeats 17–24 of Notch‐3 (entry number Q9UM47) using the rigid‐body docking servers FRODOCK (Ramírez‐Aportela et al., 2016), ZDOCK (Pierce et al., 2014), and HDOCK (Yan et al., 2020). During the docking procedure, no prior selection of potential binding sites was made. The different docking programs were employed to eliminate potential biases in the docking predictions. From each docking software, the top 50 candidate complexes of YB‐1 (YB‐1‐2G) interacting with EGF repeats were selected for further analysis. Specifically, the 150 complex structures for YB‐1 (YB‐1‐2G) were clustered based on the following criteria: (i) similar structures obtained from different docking software, (ii) modification sites 53 and 58 located at or near the binding interface, and (iii) favorable docking scores from all three docking software. This resulted in five complexes for both YB‐1 and YB‐1‐2G, which were further scrutinized using MD simulations to account for structural changes upon binding and to test their stability under more realistic conditions. For each complex, we performed three independent MD simulations of 50 ns. Additionally, we swapped YB‐1 with YB‐1‐2G and vice versa in the respective complexes and conducted another set of 3 × 50 ns MD simulations per complex. This resulted in a total of 60 MD simulations of YB‐1(−2G)/EGF 17–24 complexes, some of which were extended to 100 ns, yielding an accumulated simulation time of over 3 μs. The MD simulation settings were as described in Section 2.9.

### 4.11 NanoDSF measurements using Prometheus

The stability of YB‐1 and YB‐1‐2G was evaluated using a Nano DSF with the Prometheus NT.Plex (Nanotemper) instrument. This approach enabled the assessment of the unfolding profile of the proteins and the plotting of their fluorescence signals against temperature. The proteins were loaded into capillaries and heated from 20 to 95°C, with the fluorescence signal captured at 350 nm (Tyrosine) and 330 nm (Tryptophane).

### 4.12 Statistical analyses

Statistical analyses were carried out with GraphPad Prism 10 (GraphPad Software, La Jolla, USA). Student's *t*‐test was used to compare two experimental groups. Correlation was evaluated with Pearson's correlation coefficient. A *p* value <0.05 was considered significant. Data are shown as mean + SEM. Significance levels were as follows: * < 0.05; ** < 0.01; *** < 0.001; **** < 0.0001.

## 5 CONCLUSION

We demonstrate for the first time the connection between guanidinylation of YB‐1 (YB‐1‐2G) and its protein structure, stability, and binding to its receptor Notch‐3. Newly developed cell‐based and solid‐phase binding assays documented high‐affinity binding between ligand and receptor and identified a second binding site for YB‐1/YB‐1‐2G within Notch‐3. Furthermore, competition between YB‐1/YB‐1‐2G and the canonical ligand Jagged‐1 for potential binding to Notch‐3 was demonstrated. Finally, we identified enzymatic and biochemical processes underlying guanidinylation of YB‐1. Thus, we add another piece of the puzzle to the known mechanisms and explain how the highly conserved cold shock protein YB‐1 ensures complex multifunctionality, including intra‐ and extracellular effects through post‐translational modifications.

## AUTHOR CONTRIBUTIONS

**Anna Leitz:** Formal analysis; investigation; methodology; writing – original draft. **Batuhan Kav:** Formal analysis; visualization. **Xiyang Liu:** Methodology; investigation. **Hebah Fatafta:** Formal analysis; visualization. **Vera Jankowski:** Formal analysis; methodology. **Bastian Aggeler:** Formal analysis. **Yingying Gao:** Methodology; formal analysis; investigation. **Ina Verena Martin:** Investigation; methodology; formal analysis. **Kristian Vogt:** Formal analysis; methodology. **Rafael Kramann:** Writing – review and editing. **Tammo Ostendorf:** Writing – original draft. **Thomas Rauen:** Conceptualization; funding acquisition; writing – review and editing; data curation. **Birgit Strodel:** Conceptualization; formal analysis; supervision; writing – original draft; project administration; methodology. **Ute Raffetseder:** Conceptualization; formal analysis; supervision; funding acquisition; writing – original draft; project administration; validation; methodology.

## FUNDING INFORMATION

This work was supported by grants of the German Research Foundation (Deutsche Forschungsgemeinschaft) to UR (RA 740/8‐1, 9‐1, 11‐1), to TR (RA 1927/5‐3) to TO (OS 196/2‐1, 4‐1; SFB/TRR57 P17; KFO 5011), to VJ (TRR 219; Project‐ID 322900939, subproject S‐03; INST 948/4S‐1 FU6.6; 445703531, KFO 5011), and National Natural Science Foundation of China to XL (201806380070) and YG (202008310175) and START‐Program of the Faculty of Medicine of the RWTH Aachen University to KV (692414; 120/23).

## CONFLICT OF INTEREST STATEMENT

R.K. has no conflict of interest in regard to this work but discloses the following: he is founder and shareholder of Sequantrix GmbH, has grants from Travere Therapeutics, Galapagos, Chugai, AskBio, and Novo Nordisk, and is a consultant for or receives honoraria from Bayer, Pfizer, Novo Nordisk, Amgen, 10x Genomics, Astra Zeneca, Johnson and Johnson, Eli Lilly, Hybridize Therapeutics, and Gruenenthal. All other authors declared no competing interests.

## Supporting information


**Suppl. Fig. 1** (A/B) The specificity of the anti‐YB‐1‐2G antibody was demonstrated using a YB‐1‐2G‐specific peptide in a dot blot assay (A) and with recombinant YB‐1 (rYB‐1) that is detected only after in vitro guanidinylation shown by immunoblot with anti‐YB‐1‐2G antibody (lower panel) in comparison to total YB‐1 detected with anti‐YB‐1^C‐term^ antibody (upper panel) (B). (C)–(F) Immunoblots of MCT (C) and HK‐2 (D), (E) cell supernatants (extrac.) and cell lysates (intrac) for YB‐1, YB‐1‐2G and *β*‐Actin (as loading control). The cells were left non‐transfected (C) or were previously transfected with GATM (GATM‐OE) or control plasmid (D)–(F) and additionally stimulated with TNF‐*α* (20 ng/mL, 2 h) or left untreated. Cells from each approach were individually transfected. (G) Relationship between BUN (blood urea nitrogen) to guanidinylation mass‐signal intensity relative to the internal standard ^13^C‐ANG II in the serum of SLE patients. (H), (I) Representative MALDI‐TOF mass spectra of YB‐1‐2G after incubation with urea (100 mg/dL) with arrows indicating guanidinylated lysines 53/58 (H) and representative MALDI‐TOF/TOF fragment mass spectrum of guanidinylated peptide of YB‐1 amino acid sequence K*VIATK* (I) and VLGTVK* (J).

**Suppl. Fig. 2.** (A)–(E) Results for the five structure models of YB‐1 (left) and YB‐1‐2G (right). The most populated structure (probability between 0 and 1) in the MD simulations of the structure models are shown in the two middle columns, where the proteins are presented as cartoon with the CSD colored magenta, the N‐terminal A/P domain cyan, and the C‐terminal domain in gray. The side chains at positions 53 and 58 are shown with atomic resolution (C atoms in cyan, N in blue, O in red, H in white). The time‐averaged secondary structure propensities per residue calculated from the corresponding MD simulation are shown in the outer columns, with blue for *α*‐helix, orange for *β*‐sheet, green for turn/bend, and white for probabilities <1.0 indicating coil. Guanidinylation positions 53 and 58 are indicated by black dots at the top of each plot.

**Suppl. Fig. 3.** (A) Docking results for YB‐1 (top) and YB‐1‐2G (bottom). The five complexes obtained by protein–protein docking and applying the selection criteria (i)–(iii) (see Methods) are shown after the MD simulations. The complex structures are presented as a cartoon with the CSD colored magenta, the N‐terminal A/P domain cyan, the C‐terminal domain in gray, and the EGF 17–24 in red. **(B)** The stability of the complexes in the 3 × 50 ns MD simulations was monitored using the number of atomic contacts between YB‐1 (−2G) and EGF, where a contact is defined if the distance of any interprotein atom pair was within 0.35 nm at a given time point. In each diagram, the vertical axis is only shown in the relevant range of 2500–5000 contacts. The average over the three 3 MD runs with the standard deviation indicated as shaded region is presented for YB‐1 (orange) and YB‐1‐2G (blue). (C) The exchange between YB‐1 and YB‐1‐2G in the complexes had no effect on the stability of the complexes, as presented by the MD‐ run averaged results, using the same presentation as in (B) with orange for the replacement of YB‐1 by YB‐1‐2G and blue for the replacement of YB‐1‐2G by YB‐ 1.

**Table S1:** Docking score ranges for the top 10 complex structures per variant and docking software. For ZDOCK and FRODOCK, higher values indicated better binding modes, while for HDOCK more negative values indicate more likely binding modes.

**Table S2:** Time‐ and MD run‐averaged changes in the total number of contacts (in %) per complex, relative to the number of contacts in the starting structure obtained from docking. First, time averages were calculated for each MD run, and the mean of the three averages per complex was determined. Reported errors represent the standard errors of the mean of the time averages. The complexes predicted for YB‐1 and after modifying them to YB‐1‐2G are highlighted in yellow. Comparing the numbers for each complex shows that guanidylation did not compromise complex stability. The same holds true for the complexes predicted for YB‐1‐2G and removing the guanidylation in them, as shown in the rows highlighted in blue.

## Data Availability

The data that support the findings of this study are available from the corresponding author upon reasonable request.
