# Supplementary figures and images for: Guanidinylation of the cold shock protein YB‐1: Molecular basis, structural changes and Notch‐3 receptor binding
Source: Protein Sci. 2025 Jun 25;34(7):e70188. doi: 10.1002/pro.70188 (PMC12198050; doi:10.1002/pro.70188)

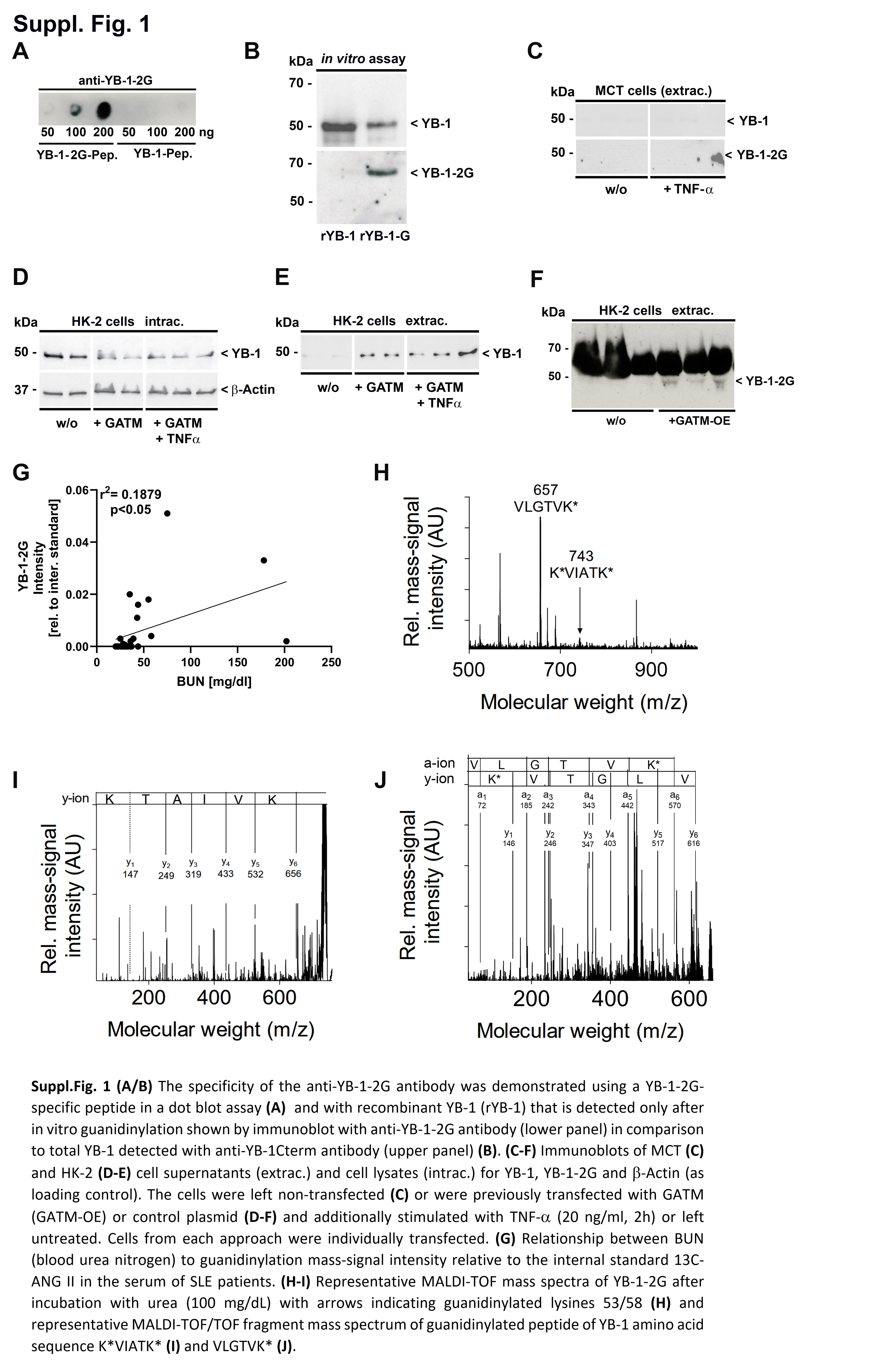

Supplement: Supplementary file 1 — Suppl. Fig. 1 (A/B) The specificity of the anti‐YB‐1‐2G antibody was demonstrated using a YB‐1‐2G‐specific peptide in a dot blot assay (A) and with recombinant YB‐1 (rYB‐1) that is detected only after in vitro guanidinylation shown by immunoblot with anti‐YB‐1‐2G antibody (lower panel) in comparison to total YB‐1 detected with anti‐YB‐1C‐term antibody (upper panel) (B). (C)–(F) Immunoblots of MCT (C) and HK‐2 (D), (E) cell supernatants (extrac.) and cell lysates (intrac) for YB‐1, YB‐1‐2G and β‐Actin (as loading control). The cells were left non‐transfected (C) or were previously transfected with GATM (GATM‐OE) or control plasmid (D)–(F) and additionally stimulated with TNF‐α (20 ng/mL, 2 h) or left untreated. Cells from each approach were individually transfected. (G) Relationship between BUN (blood urea nitrogen) to guanidinylation mass‐signal intensity relative to the internal standard 13C‐ANG II in the serum of SLE patients. (H), (I) Representative MALDI‐TOF mass spectra of YB‐1‐2G after incubation with urea (100 mg/dL) with arrows indicating guanidinylated lysines 53/58 (H) and representative MALDI‐TOF/TOF fragment mass spectrum of guanidinylated peptide of YB‐1 amino acid sequence K*VIATK* (I) and VLGTVK* (J). [file PRO-34-e70188-s002.jpg]

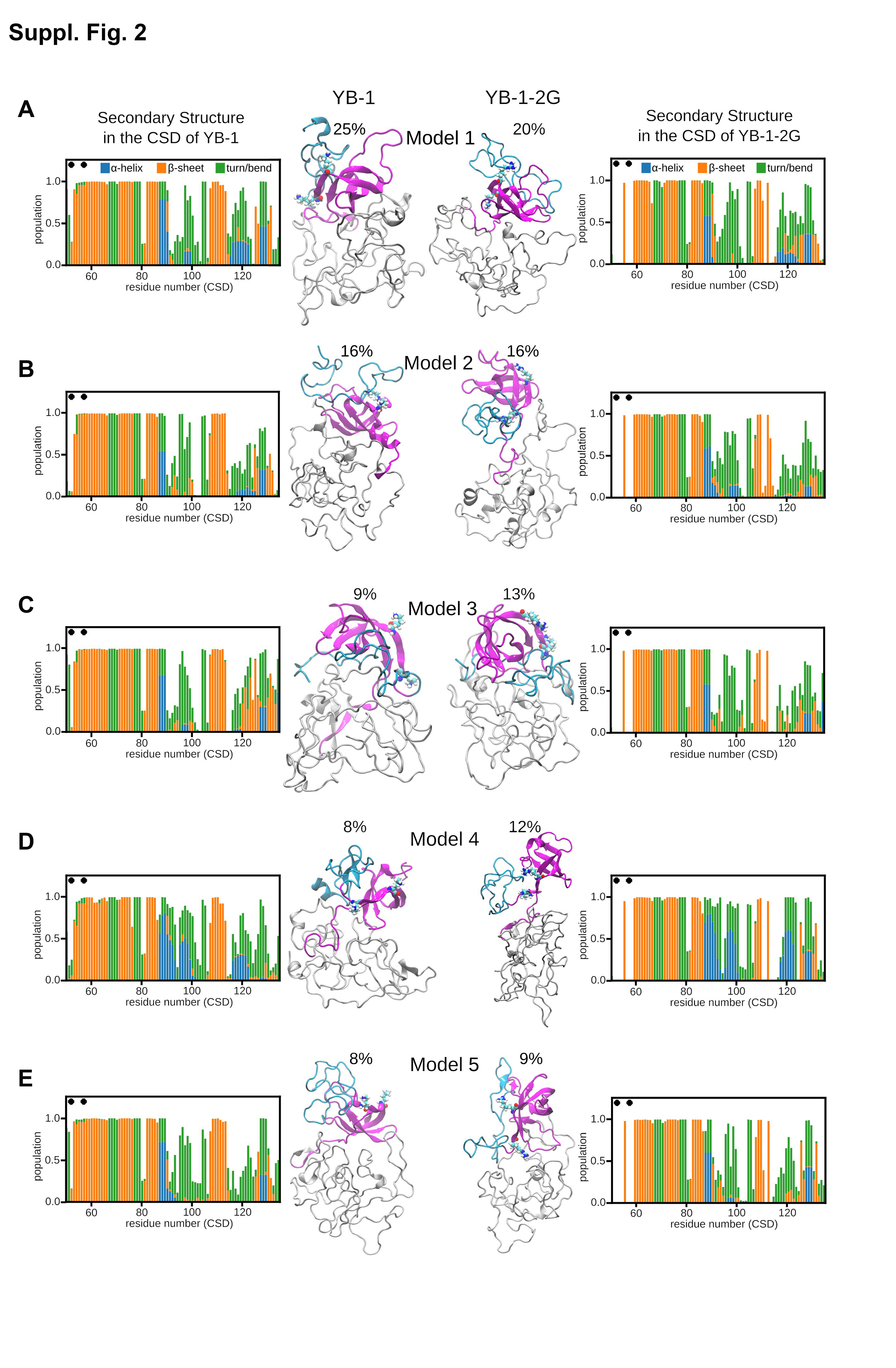

Supplement: Supplementary file 2 — Suppl. Fig. 2. (A)–(E) Results for the five structure models of YB‐1 (left) and YB‐1‐2G (right). The most populated structure (probability between 0 and 1) in the MD simulations of the structure models are shown in the two middle columns, where the proteins are presented as cartoon with the CSD colored magenta, the N‐terminal A/P domain cyan, and the C‐terminal domain in gray. The side chains at positions 53 and 58 are shown with atomic resolution (C atoms in cyan, N in blue, O in red, H in white). The time‐averaged secondary structure propensities per residue calculated from the corresponding MD simulation are shown in the outer columns, with blue for α‐helix, orange for β‐sheet, green for turn/bend, and white for probabilities <1.0 indicating coil. Guanidinylation positions 53 and 58 are indicated by black dots at the top of each plot. [file PRO-34-e70188-s003.jpg]

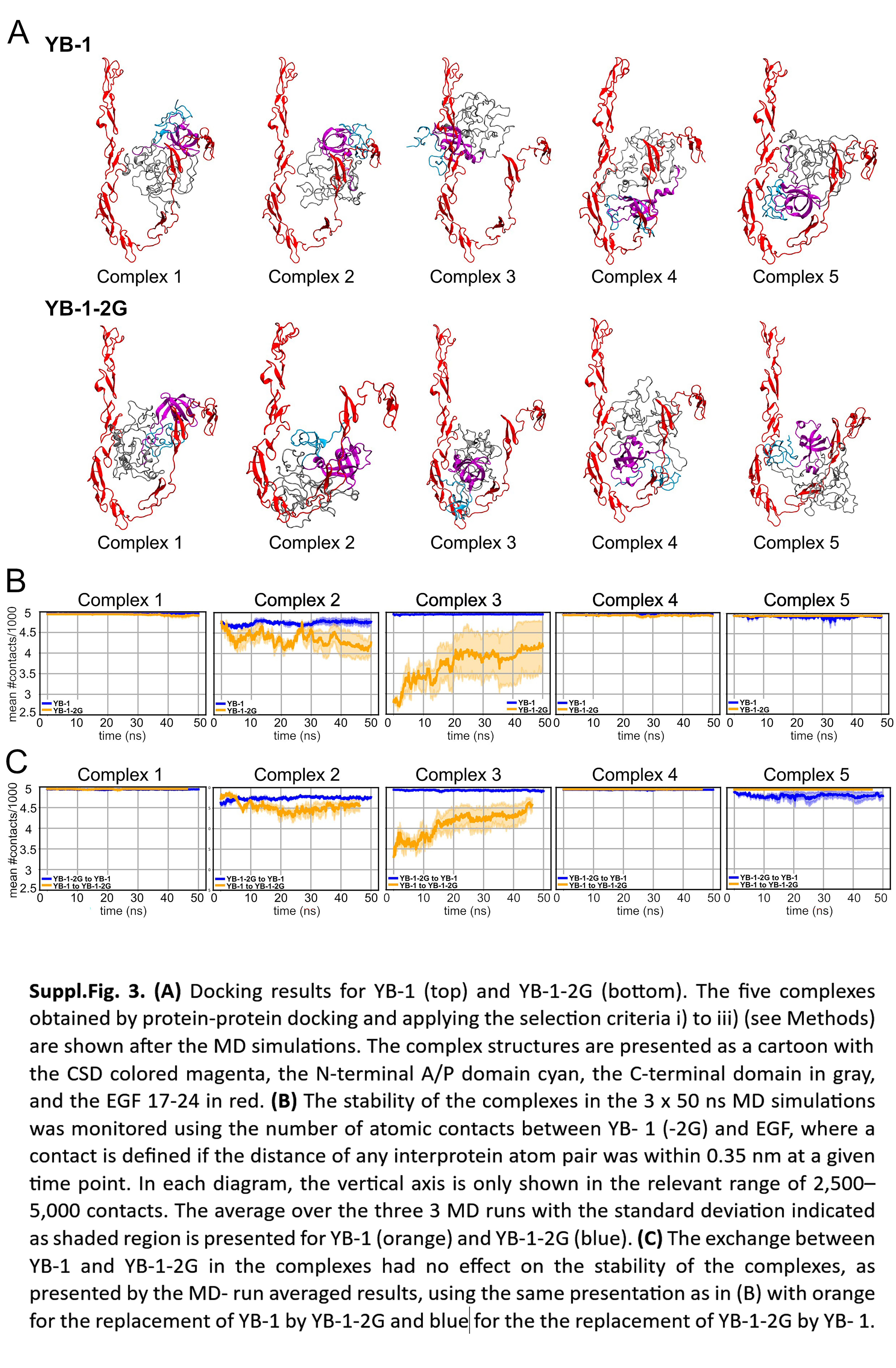

Supplement: Supplementary file 3 — Suppl. Fig. 3. (A) Docking results for YB‐1 (top) and YB‐1‐2G (bottom). The five complexes obtained by protein–protein docking and applying the selection criteria (i)–(iii) (see Methods) are shown after the MD simulations. The complex structures are presented as a cartoon with the CSD colored magenta, the N‐terminal A/P domain cyan, the C‐terminal domain in gray, and the EGF 17–24 in red. (B) The stability of the complexes in the 3 × 50 ns MD simulations was monitored using the number of atomic contacts between YB‐1 (−2G) and EGF, where a contact is defined if the distance of any interprotein atom pair was within 0.35 nm at a given time point. In each diagram, the vertical axis is only shown in the relevant range of 2500–5000 contacts. The average over the three 3 MD runs with the standard deviation indicated as shaded region is presented for YB‐1 (orange) and YB‐1‐2G (blue). (C) The exchange between YB‐1 and YB‐1‐2G in the complexes had no effect on the stability of the complexes, as presented by the MD‐ run averaged results, using the same presentation as in (B) with orange for the replacement of YB‐1 by YB‐1‐2G and blue for the replacement of YB‐1‐2G by YB‐ 1. [file PRO-34-e70188-s001.jpg]
