# Supplementary material for: Guanidinylation of the cold shock protein YB‐1: Molecular basis, structural changes and Notch‐3 receptor binding
Source: Protein Sci. 2025 Jun 25;34(7):e70188. doi: 10.1002/pro.70188 (PMC12198050; doi:10.1002/pro.70188)
Supplement: Supplementary file 4 — Table S1: Docking score ranges for the top 10 complex structures per variant and docking software. For ZDOCK and FRODOCK, higher values indicated better binding modes, while for HDOCK more negative values indicate more likely binding modes. [file PRO-34-e70188-s004.docx]

|  | **YB-1/EGF 17–24** | **YB-1-2G/EGF 17–24** |
| --- | --- | --- |
| **ZDOCK** | 1335 – 1585 | 1335 – 1571 |
| **FRODOCK** | 3380 – 4580 | 3540 – 4765 |
| **HDOCK** | (–340) – (–260) | (–320) – (–285) |

**Table S1:** Docking score ranges for the top 10 complex structures per variant and docking software. For ZDOCK and FRODOCK, higher values indicated better binding modes, while for HDOCK more negative values indicate more likely binding modes.
