# Supplementary material for: Guanidinylation of the cold shock protein YB‐1: Molecular basis, structural changes and Notch‐3 receptor binding
Source: Protein Sci. 2025 Jun 25;34(7):e70188. doi: 10.1002/pro.70188 (PMC12198050; doi:10.1002/pro.70188)
Supplement: Supplementary file 5 — Table S2: Time‐ and MD run‐averaged changes in the total number of contacts (in %) per complex, relative to the number of contacts in the starting structure obtained from docking. First, time averages were calculated for each MD run, and the mean of the three averages per complex was determined. Reported errors represent the standard errors of the mean of the time averages. The complexes predicted for YB‐1 and after modifying them to YB‐1‐2G are highlighted in yellow. Comparing the numbers for each complex shows that guanidylation did not compromise complex stability. The same holds true for the complexes predicted for YB‐1‐2G and removing the guanidylation in them, as shown in the rows highlighted in blue. [file PRO-34-e70188-s005.docx]

**Suppl Table 2:**

|  | **Complex 1** | **Complex 2** | **Complex 3** | **Complex 4** | **Complex 5** |
| --- | --- | --- | --- | --- | --- |
| **YB-1** | -0.22 ± <0.01 | -9.12 ± 0.01 | 33.18 ± 0.03 | -0.20 ± <0.01 | -0.04 ± <0.01 |
| **YB-1 to YB-1-2G** | -0.08 ± <0.01 | -4.17 ± <0.01 | 24.18 ± 0.02 | 0.01 ± <0.01 | -0.01 ± < 0.01 |
| **YB-1-2G** | -0.01 ± < 0.01 | 0.03 ± <0.01 | -0.07 ± <0.01 | -0.03 ± <0.01 | -0.66 ± <0.01 |
| **YB-1-2G to YB-1** | -0.12 ± <0.01 | 2.36 ± <0.01 | -0.33 ± <0.01 | -0.05 ± <0.01 | -1.83 ± <0.01 |

**Table S2:** Time- and MD run-averaged changes in the total number of contacts (in %) per complex, relative to the number of contacts in the starting structure obtained from docking. First, time averages were calculated for each MD run, and the mean of the three averages per complex was determined. Reported errors represent the standard errors of the mean of the time averages. The complexes predicted for YB-1 and after modifying them to YB-1-2G are highlighted in yellow. Comparing the numbers for each complex shows that guanidylation did not compromise complex stability. The same holds true for the complexes predicted for YB-1-2G and removing the guanidylation in them, as shown in the rows highlighted in blue.
